# Acute Disruption of Bone Marrow B Lymphopoiesis and Apoptosis of Transitional and Marginal Zone B Cells in the Spleen following a Blood-Stage *Plasmodium chabaudi* Infection in Mice

**DOI:** 10.1155/2011/534697

**Published:** 2011-05-04

**Authors:** Viki Bockstal, Nathalie Geurts, Stefan Magez

**Affiliations:** ^1^Laboratory for Cellular and Molecular Immunology, Vrije Universiteit Brussel, Pleinlaan 2, 1050 Brussels, Belgium; ^2^Department of Molecular and Cellular Interactions, VIB, Brussels, Belgium; ^3^Laboratory of Immunobiology, Rega Institute for Medical Research, University of Leuven, Minderbroedersstraat 10, 3000 Leuven, Belgium

## Abstract

B cells and antibodies are essential for the protective immune response against a blood-stage *Plasmodium* infection. Although extensive research has focused on memory as well as plasma B-cell responses during infection, little is known about how malaria affects B-cell development and splenic maturation into marginal zone B (MZB) and follicular B (FoB) cells. In this study, we show that acute *Plasmodium chabaudi* AS infection in C57Bl/6 mice causes severe disruption of B lymphopoiesis in the bone marrow, affecting in particular pro-, pre-, and immature B cells as well as the expression of the bone marrow B-cell retention chemokine CXCL12. In addition, elevated apoptosis of transitional T2 and marginal zone (MZ) B cells was observed during and subsequent to the control of the first wave of parasitemia. In contrast, Folllicular (Fo) B cells levels were retained in the spleen throughout the infection, suggesting that these are essential for parasite clearance and proper infection control.

## 1. Introduction

Malaria is a major health problem in developing countries, affecting each year at least 300–500 million individuals of which more than 1 million people die of serious complications. Primarily in children beyond 5 years, parasite-mediated processes and excessive or uncontrolled inflammation cause malaria pathogenesis, characterized by severe malarial anemia (SMA), cerebral malaria (CM), malaria-associated acute lung injury (ALI) and its more severe form malaria-associated acute respiratory distress syndrome (MA-ARDS) [1]. 

During a malaria infection, pre-erythrocytic liver stages are mainly attacked by CD8^+^ effector cells and IFN-*γ*, whereas antibodies are key components against the asexual blood stage of the *Plasmodium* life cycle [1, 2]. Studies in mice lacking B cells revealed that they were unable to clear a *Plasmodium chabaudi *AS (*Pc*AS) infection and instead displayed chronic unresolved parasitemia levels for periods as long as 120 days [3]. Hence, B cells and malaria specific antibodies are, in addition to CD4^+^T cells, required for effective antimalarial immunity [4–6]. Passive serum transfer studies in human corroborate these findings [7, 8]. Despite the key role of antibodies in immunity to malaria, there is a gap in our knowledge on the cellular basis of these humoral responses during infection. In malaria, several experimental studies report that a single *Pc*AS infection induces both short-lived and long-lived plasma cells including the generation of functional memory B-cells [9–11]. In addition, some recent human malaria studies report the generation of long-lived plasma cells and memory B-cells, whether or not in conditions of frequent re-exposure. However, Wykes et al. revealed that *Plasmodium yoelii *is capable of destroying vaccine-induced memory [12], suggesting defects in the B-cell lineage might be induced during a malaria infection. 

B-cell development of cells of the B2 lineage under normal conditions occurs via a series of bone marrow (BM) stromal cell-facilitated processes that begin within the hematopoietic stem cell pool and proceed in hierarchical steps of lineage commitment [13, 14]. B lymphopoiesis yields several developmental stages of pre-pro-B, pro-B, pre-B and eventually immature B cells, which show a high expression of the IgM form of the antigen receptor and low or no expression of the IgD maturation marker [15, 16]. To complete their development, immature B cells migrate through the periphery; however, only 10% reaches the spleen as transitional B cells of the T1 type. Important is the fact that under inflammatory immune conditions, BM lymphopoiesis is often severely reduced and is compensated for by a splenic cell differentiation process that involves the same B-cell differentiation steps, referred to as extramedullary lymphopoiesis [17, 18]. Once the transitional T1 stage has been reached, B cells develop further into T2 transitional B cells that in turn can mature into either Marginal Zone B (MZB) cells or Follicular B (FoB) cells [19]. Each of these populations is distinguished by a unique set of cell surface antigens, allowing monoclonal antibody (mAb) phenotyping by multicolor flow cytometry [20–22].

Although several studies have focused on analyzing the T- and B-cell memory response in murine models for malaria as well as the plasma B-cell response, little is known about how a *Plasmodium* infection affects the process of B lymphopoiesis in the bone marrow and the maturation into naive, resting cells in the spleen. Recently, it has been reported that acute *Pc*AS infection induces the transient depletion of functional myeloid-erythroid progenitors and loss of common lymphocyte progenitors (CLPs), which under normal circumstances sustain both T- and B-cell development [23]. Because of the essential protective role for B cells in blood-stage malaria, it is important to understand the kinetics and regulation of the whole B-cell cycle from development to differentiation into plasma and memory B cells during infection. In this study, we demonstrate that during the acute phase of a *Pc*AS infection B lymphopoiesis is severely compromised in the bone marrow and infection-induced apoptosis of T2 and MZB cells depletes these populations from the spleen. The retention of splenic FoB cells in the spleen, being a source for high-affinity, parasite-specific-class-switched plasma B-cell responses, is most likely essential for the proper parasitemia clearance following the acute phase of infection. During the chronic phase of infection, when parasitemia levels are low and controlled, B lymphopoiesis in the bone marrow and maturation into transitional T2 and MZB cells in the spleen recovers completely and most likely this is crucial for the further control of infection.

## 2. Material and Methods

### 2.1. Parasites and Infection in Mice

Male C57BL/6 wild type (Janvier, Le Genest Saint-Isle, France; 6–8 weeks old) were infected by intraperitoneal (i.p.) injection of blood containing 10^4^ *Pc*AS infected red blood cells (iRBC) (a kind gift of the late Professor Dr. D. Walliker, University of Edingburgh, Scotland, UK). Parasitemia was analyzed using Giemsa staining on a blood smear collected from the tail vein during infection. All mice were housed under barrier conditions and all experimental animal procedures were approved by the appropriate university's ethics committees.

### 2.2. Cell Isolation and Flow Cytometric Analysis

B-cell populations were analyzed by flowcytometry. Both spleen and bone marrow from femur and tibia were harvested from noninfected control and *Pc*AS infected mice 10, 20, 30 and 41 days postinfection (pi). Cell suspensions were prepared in FACS buffer (1.0% BSA (Sigma, St. Louis, MO) in DPBS) and red blood cells were lysed using lysis buffer (0.15 M NH4Cl, 1.0 mM KHCO3, 0.1 mM Na2-EDTA). Nonspecific binding sites were blocked using Fc block (CD16/CD32 Fc*γ* III/II, BD biosciences, Erembodegem, Belgium) for 30 minutes at 4°C. Cells were washed twice with FACS buffer and stained with biotin- or fluorochrome-conjugated primary antibodies (Section 2.3) for 30 minutes at 4°C. After washing twice, cell suspensions stained with biotin-conjugated antibodies were incubated with streptavidin-conjugated fluorochromes. which detects cell bound biotinylated antibodies, and incubated for an additional 30 minutes at 4°C. Finally, cells were resuspended in FACS buffer with 1 *μ*g of 7-amino-actinomycin D (7AAD), a fluorescent DNA dye that binds to membrane permeable dead or dying cells, (BD biosciences). Analyses were performed using a FACS Canto II flow cytometer (BD Biosciences) and data were processed using FLOWJO software (Tree Star Inc., Ashland, OR). The total number of cells in each population was determined by multiplying the percentages of subsets within a series of marker negative or positive gates by the total cell number determined for each tissue. 

### 2.3. Antibodies and Detection Reagents

The following antibodies were added to 100 *μ*L aliquots of 10^6^ Fc-blocked leukocytes prepared as described above: 0.5 *μ*g anti-CD23-FITC (clone B3B4), 0.5 *μ*g anti-IL7r*α*-FITC (clone A7R34), 0.5 *μ*g anti-CD11b-FITC (clone M1/70; 0.5 mg/mL), 0.5 *μ*g anti-CD45R (B220)-FITC (clone RA3-6B2), 0.2 *μ*g anti-IgM-PE (clone II/41), 0.2 *μ*g anti-CD93–PE (clone AA4.1), 0.5 *μ*g anti-CD95-FITC (clone Jo2), 0.25 *μ*g hamster IgG2, *κ* isotype control (clone B81-3), 0.2 *μ*g anti-IgM PE-Cy7 (clone II/41), 0.2 *μ*g of anti-CD45R (B220)-PE-Cy7 (clone RA3-6B2), 0.2 *μ*g of anti-CD93-APC (clone AA4.1), and 0.2 *μ*g of anti-CD117 (ckit)-APC (clone 2B8), purchased from eBioscience (San Diego, CA); 0.2 *μ*g anti-CD1d-PE (clone 1b1), 0.2 *μ*g of anti-CD43-PE (clone 1B11), 0.2 *μ*g of anti-CD45R (B220)-APC-Cy7 (clone RA3-6B2), 0.2 *μ*g of anti-CD19-APC-Cy7 (clone 1D3), 0.2 *μ*g of streptavidin-PerCP, and 0.2 *μ*g of streptavidin-PE-Texas Red, purchased from BD Biosciences (Erembodegem, Belgium); 2 *μ*g of each of the following antibodies: CD3*ε*, CD11b (Mac-1), Gr-1 (Ly-6G and Ly-6C) and Ter-119 (Ly-76) from the Biotin-conjugated Mouse Lineage Panel (BD Biosciences, Erembodegem, Belgium).

### 2.4. Flow Cytometric Analyses of Apoptosis

Cells were stained as described in Section 2.3 with antibodies. For the polycaspases-based apoptosis assay, labeled cells were further reacted with the FLICA fluorescent inhibitor of caspase-1, -3, -4, -5, -6, -7, -8, and -9, using the FAM Poly Caspases Assay Kit for flow cytometric analysis (Molecular probes, Invitrogen, Merelbeke, Belgium).

### 2.5. Statistical Analysis

Statistical comparisons were performed by *t*-test or one way ANOVA and means were compared using Tukey and Dunnett's post test when *P* ≤ .05 (GraphPad Prism v.4.0, GraphPad Software Inc. San Diego, CA).

## 3. Results

### 3.1. *Pc*AS Infection Causes a Transient Suppression of B-Cell Development in the Bone Marrow

**Figure 1:**
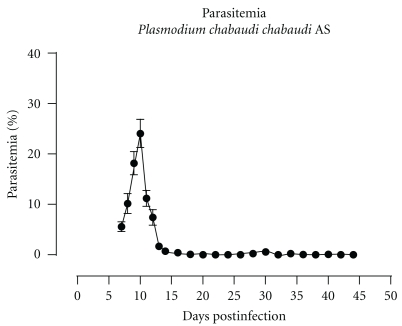
Parasitemia profile of *Pc*AS infection. Course of malaria infection in C57Bl/6 mice infected with blood stages of *Pc*AS. Data are representative of at least 3 independent experiments with *n* > 10 for each experiment (mean ± SEM).

**Figure 2:**
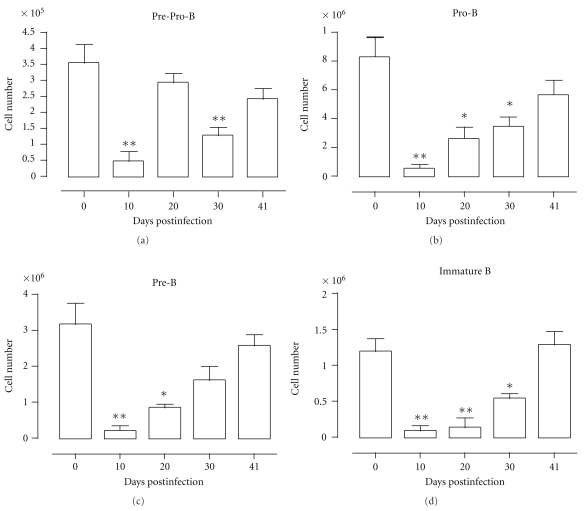
B dyslymphopoiesis in bone marrow during *Pc*AS infection. Bone marrow cells from uninfected mice and mice infected with *Pc*AS for 10–41 days were stained for surface markers commonly used to define developing B cells and analyzed using FACS. Data are represented as mean of three mice per group ± SEM, two independent repeat experiments were performed and statistics are compared to uninfected controls (*) *P* < .05, (**) *P* < .01.

To evaluate the kinetics of the different B-cell precursor populations in the bone marrow during *Pc*AS malaria, total bone marrow and spleen cells were isolated from uninfected control mice and mice at 10, 20, 30, and 41 days pi and analyzed using flowcytometry according to Table 1. Parasitemia was measured as percentage of parasitized red blood cells throughout infection and the representative profile is shown in Figure 1. At day 10 of infection, when parasitemia levels are about to peak, there was a more than 95% depletion of all B-cell specific precursor populations in the bone marrow. Afterwards, B-cell lymphopoiesis slowly recovered until pre-pro-B, pro-B and pre-B cell numbers are back to preinfection levels at day 41 pi (Figure 2). 

**Figure 3:**
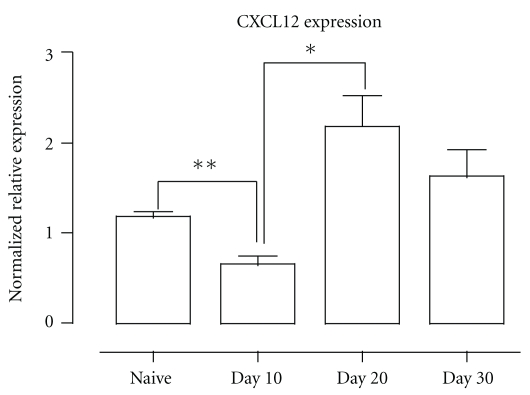
Bone marrow CXCL12 mRNA expression during *Pc*AS infection. BM was isolated and amplified using intron-spanning primers specific for CXCL12 via Quantitative PCR. Data were normalized to S12 expression and are presented as relative expression compared to uninfected controls. Data are represented as mean of 3 mice ± SEM and are representative of two separate experiments were performed. (*) *P* < .05, (**) *P* < .01.

The interaction between the chemokine CXCL12 and its receptor CXCR4 on developing B cells is crucial for B lymphopoiesis [14, 24], eliciting a stepwise progression of developing B cells through specialized bone marrow niches [25]. Here, we observed that a *Pc*AS infection caused a significant reduction in CXCL12 expression in the bone marrow on day 10 pi, which correlated with the observed reduction in B-cell precursor cells at that time point. At days 20 and 30 of infection, a significant recovery in CXCL12 expression was measured in the bone marrow, coinciding with the observed slow recovery of B lymphopoiesis (Figure 3).Furthermore, when we analyzed the developing B cells in the bone marrow of infected mice for apoptosis induction, there was no difference with uninfected control mice (data not shown), suggesting that apoptosis is not a major contributor to the observed depletion of B-cell precursor populations in the spleen. 

**Figure 4:**
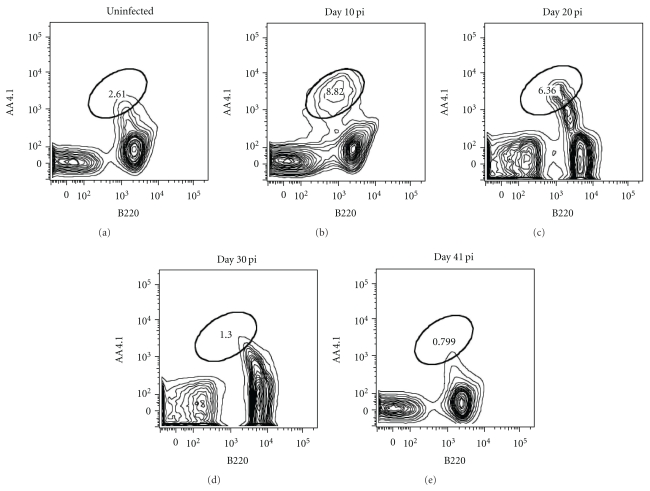
Extramedullary B lymphopoiesis in spleen during *Pc*AS infection. Spleens cells from noninfected mice and mice infected with *Pc*AS for 10–41 days were stained for surface markers used to define developing B cells. The total B cell precursor population, including pre-pro-B, pro-B, pre-B and immature B, was detected as 7AAD^−^Lin^−^B220^int^AA4.1^hi^ using FACS. Data are representative of two separate experiments.

Inflammation, characterized by high levels of TNF and other type 1 cytokines, has a negative effect on bone marrow B-cell development. Induction of a reduced CXCL12 expression followed by migration of developing B cells out of the bone marrow has been described to coincide with the occurence of extramedullary B lymphopoiesis in the spleen [17, 18]. Here, on day 10 of a *Pc*AS infection, a distinct population of developing B cells (pre-pro-, pro-, pre- and immature B cells defined here together as 7AAD^−^, Lin^−^, AA4.1^hi^, B220^+^) was found in the spleen, which was significantly increased compared to uninfected controls (Figure 4). However, the number of developing B cells in the spleen had diminished already by day 20 pi and this population had disappeared completely by day 30 pi (Figure 4), which coincided with the observed recovery of both CXCL12 expression (Figure 3) and B-cell development in the bone marrow at that time (Figure 2). 

### 3.2. *Pc*AS Malaria Results in Apoptosis of Transitional B Cells in the Spleen

**Figure 5:**
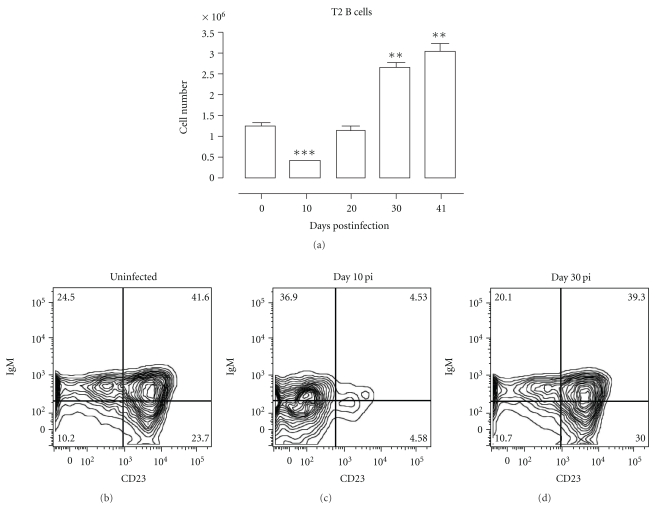
Depletion of transitional type 2 B cells in spleen during *Pc*AS infection. (a) Spleens cells from noninfected mice and mice infected with *Pc*AS for 10–41 days were stained for surface markers commonly used to define transitional T2 B cells and analyzed using FACS. Data are represented as mean of three mice per group ± SEM, two independent repeat experiments were performed and statistics are compared to uninfected controls (**) *P* < .01, (***) *P* < .001. (b), (c), and (d) Transitional T2 B cells were detected as (AA4.1^+^B220^+^) IgM^+^CD23^+^ in uninfected mice and mice on day 10 and 30 pi.

Transitional B cells form the cellular link between the B-cell development in the bone marrow and the maturation into mature marginal zone or follicular B cells in the spleen. During *Pc*AS infection in mice, a significant decrease of T2 transitional B cells was observed on day 10 of infection in the spleen, followed by a complete recovery on day 20 pi. (Figure 5). 

**Figure 6:**
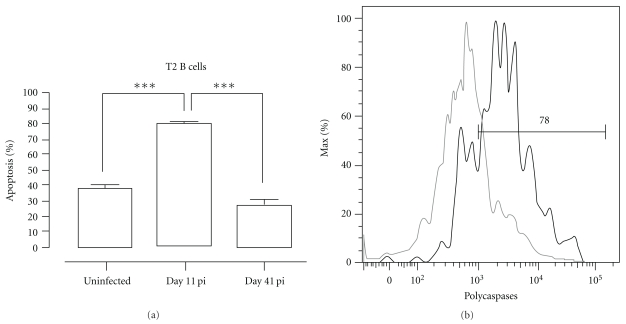
*Pc*AS infection-induced apoptosis of transitional B cells. Spleens cells from uninfected control mice and mice infected with *Pc*AS were stained for surface markers commonly used to define transitional T2 B cells and the amount of active caspase 1, -3, -4, -5, -6, -7, -8 and -9 was measured intracellular by flowcytometry. (a) Percentage of apoptotic cells within T2 transitional B-cell population in uninfected controls versus infected mice on day 11 and 41 pi. Data are represented as mean of three mice per group ± SEM (***) *P* < .001 and representative of two separate experiments. (b) Representative histogram of uninfected controls (grey line) versus a day 11 pi (black line).

The induction of apoptosis was analyzed by measuring the amount of active caspases inside the cell using flow cytometry. Interestingly, about 80% of the T2 transitional B-cell populations was triggered to undergo apoptosis on day 11 pi. As a control, on day 41 of infection when T2 B-cell numbers are no longer depleted, the level of apoptosis induction had returned to preinfection level (Figure 6).

### 3.3. *Pc*AS Infection Causes Apoptosis of Marginal Zone (MZ) B Cells But Not Follicular (Fo) B Cells

**Figure 7:**
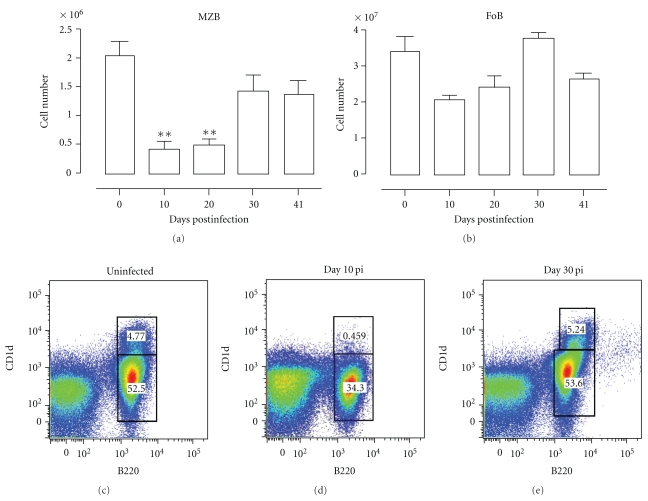
Depletion of MZB but not FoB cells in spleen during *Pc*AS infection. (a) and (b) Spleens cells from noninfected mice and mice infected with *Pc*AS for 10–41 days were stained for surface markers commonly used to define MZB (a) and FoB (b) cells and analyzed using FACS. Data are represented as mean of three mice per group ± SEM, two independent repeat experiments were performed and statistics are compared to uninfected controls (**) *P* < .01. (c), (d), and (e) MZB and FoB cells were detected as (AA4.1^−^) B220^+^CD1d^+^ (CD23^lo^CD21^hi^), respectively, (AA4.1^−^) B220^+^CD1d^−^ (CD23^hi^CD21^lo^), in noninfected mice and mice on day 10 and 30 pi.

Analysis of mice infected with *Pc*AS revealed that there was a more than 95% depletion in MZB cells at days 10 and 20 of infection in the spleen, followed by a strong recovery on day 30 pi. In contrast, there was no significant decrease in FoB cell numbers during the course of infection (Figure 7). 

**Figure 8:**
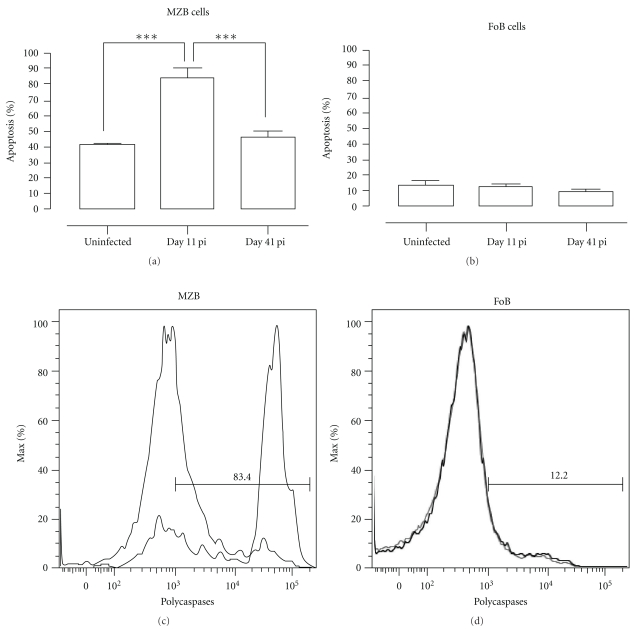
*Pc*AS infection-induced apoptosis of MZB but not FoB cells. Spleens cells from noninfected mice and mice infected with *Pc*AS for 11 and 41 days were stained for surface markers commonly used to define MZB and FoB cells and the amount of active caspase 1, -3, -4, -5, -6, -7, -8, and -9 was measured intracellular by flowcytometry. (a) and (b) Percentage of apoptotic cells within MZB or FoB cell population in uninfected controls versus infected mice on day 11 and 41 pi. Data are represented as mean of three mice per group ± SEM (***) *P* < .001. (c) and (d) Representative histogram of uninfected controls (grey line) versus a day 11 pi (black line).

Further analysis revealed that about 80% of the MZB cells present in the spleen on day 11 of infection was undergoing apoptosis (Figure 8). By day 41 of infection the level of caspase activation had returned to preinfection levels, which coincided with the observed recovery in MZB cell numbers in the spleen at that time. In contrast, there was no increased induction of FoB apoptosis during a *Pc*AS infection. 

## 4. Discussion

Polyclonal lymphocyte activation associated with splenomegaly, hypergamma-globulinemia and autoantibody production are common features of *Plasmodium *infections in both humans and experimental murine models. *Pc*AS infections in mice are widely used as a model for *P. falciparum *infections in humans. A primary infection is characterized by parasitemia levels between 20–30% around day 10 of infection, followed by a 2- to 3-month low-grade chronic infection. Hence, this is an appropriate model to study the immunological processes underlying the acquisition of semi-immunity following a malaria infection [9]. Strong innate and Th1 responses are related to the early stage of infection and protection is believed to be associated with IFN-*γ*, TNF and NO production. Around peak parasitemia a switch to Th2 cytokine production occurs and the decline in acute primary parasitemia and chronic stages of the infection is controlled primarily in a B-cell and antibody-dependent way [26–28]. The protective function of antibodies during blood stage *Plasmodium* infection stretches from being involved in the opsonization of merozoites and parasitized erythrocytes to the stimulation of macrophage and neutrophil phagocytosis, parasite sequestration, and direct merozoite neutralization [29, 30]. There is strong evidence that naturally acquired immunity to blood-stage malaria is strongly dependent on antibodies [29, 31–33]. However, data from both experimental murine and human malaria show loss of activated or memory CD4^+^T cells, B cells and plasma cells and short-lived malaria-specific antibodies after a primary acute infection [28, 34–36]. Hence, it is possible that the lack of efficient, long lasting protective immunity observed in human malaria is due to defects in the B-cell lineage. Immunization and infection with *Plasmodium* or other pathogens have already been described to transiently suppress and/or alter bone marrow hematopoiesis, followed by an increased migration of immature cells out of the bone marrow, including RAG+ immature B cells [37]. In this context, an increase in GL7-expressing cells was detected in PBMC of *Pc*AS infected mice and these cells also appeared to be mainly immature B cells which had presumably migrated from the bone marrow [38]. In agreement with this, we show in this study that during an acute primary blood stage *Pc*AS infection, B-cell development is seriously impaired with a more than 95% depletion of B-cell precursor cells in the bone marrow. Later, when the acute first peak of infection has been overcome and parasitemia remains below detection levels, B lymphopoiesis slowly but surely recovers in the bone marrow. Loss of developing B cells from the bone marrow has been correlated before with a reduction in bone marrow CXCL12, resulting in a mobilization of developing B cells to the periphery. In accordance with this, we report here both a reduced CXCL12 production in the bone marrow and an influx of developing B cells in the spleen during the acute phase of infection. This premature migration out of the bone marrow may reflect the physiologic process for replenishing the transitional B-cell pool in the spleen, but it actually results in a transient loss of bone marrow B lymphopoiesis. The inflammatory cytokine TNF has been reported to modulate B-cell development by reducing the ability of the bone marrow to retain developing B cells. ([17], reviewed in [18]). In the serum of *Pc*AS-infected mice, TNF levels are very high around peak parasitemia [39], so it is possible that a TNF-modulated reduction in CXCL12 production in the bone marrow is contributing to the observed impaired B lymphopoiesis. 

It had already been described before that the majority of apoptotic cells in the spleen of *Pc*AS infected mice are B cells, most likely comprising polyclonally activated B cells through a mechanism that could possibly involve Fas [40]. Here, we show that a *Pc*AS infection causes a severe depletion of transitional T2 B cells and MZB cells in the spleen but there is no significant change in FoB cell numbers during the early stage of infection. The loss of transitional T2 and MZB cells mainly results from the highly increased occurrence of apoptosis in this population as about 80% of all T2 and MZB cells are undergoing apoptosis in the acute phase of infection. However, most likely MZB cells also serve as an important source for T-cell independently generated IgM+ plasma B cells during the early stage of infection, seen that parasite-specific IgM responses are already high around day 8 and peak at day 14 of infection [41]. In this context, it can be speculated that by inducing massive MZB apoptosis, the parasite is playing in its own advantage since protection during an acute *Pc*AS infection is (partially) dependent on specific IgM and not natural IgM (mostly produced by B-1 cells) [40]. In contrast, there is no infection-induced apoptosis of FoB cells in the spleen. During *Pc*AS infection in mice, there are many class-switched plasma B cells found in both peripheral blood and spleen, comprising mostly IgG2a-secreting cells [42–45] that play an important role in parasite clearance. Most likely the conservation of the FoB cell pool during infection, being the major source for high-affinity malaria-specific class-switched plasma B cells, which in turn are essential for parasite clearance, allows the mouse to control the *Pc*AS infection.

It is believed that periodic reinfection is necessary to maintain acquired immunity to malaria and that *Plasmodium*-specific antibodies are short lived in the absence of reinfection, implying that the B-cell memory response to malaria infection may be defective or suboptimal [1, 46]. However, the development and longevity of malaria-specific B-cell memory is widely debated. Some studies have shown that malaria infection interferes with the development of long-lived plasma cells and memory B cells [35, 47, 48], while others have demonstrated in both human infections and animal models that isotype-switched memory B cells do develop and are detectable for months or years in blood and/or spleen [38, 46, 49]. However, persistence of memory B cells in peripheral blood alone may not correlate with longevity of specific humoral immunity. In this context, in both malaria patients as well as murine models of malaria, both short-lived [50, 51] and long-lived [49] anti-*Plasmodium* antibody responses have been reported in longitudinal and cross-sectional studies. However, in the case of *Plasmodium yoelii*, infection-induced deletion of vaccine-specific memory B cells as well as long-lived plasma cells including those specific for bystander immune responses have been described [12]. Hence, it can be speculated that the prospects for developing an effective vaccine that is protective against human *Plasmodium falciparum* or *Plasmodium vivax* infections are much better than for African trypanosome infections, if further research clarifies for several different infection settings whether there is no continuous destruction of immunological memory during malaria infections or whether there is a situation similar to the one observed for African trypanosome infections [52]. 

Taken together, our observations fit well with previous studies and elucidate in detail the kinetics of B-cell development and maturation during malaria infection, which is essential for understanding the disease and the future development of antimalaria vaccines.

## Figures and Tables

**Table 1:** Differentiation antigen phenotypes of developing and mature B2 B cells.

Cell population	Suface marker phenotype
pre-pro-B	Lin^−^ (Ter119, CD3*ε*, CD11b, Gr1, NK1.1), B220^+^, AA4.1^+^, IgM^−^, CD19^−^, CD43^hi^
pro-B	Lin^−^ (Ter119, CD3*ε*, CD11b, Gr1, NK1.1), B220^+^, AA4.1^+^, IgM^−^, CD19^+^, CD43^hi^
pre-B	Lin^−^ (Ter119, CD3*ε*, CD11b, Gr1, NK1.1), B220^+^, AA4.1^+^, IgM^−^, CD19^+^, CD43^lo/−^
immature B	Lin^−^ (Ter119, CD3*ε*, CD11b, Gr1, NK1.1), B220^+^, AA4.1^+^, IgM^+^, CD19^+^, CD43^lo/−^
T1 transitional	B220^+^, AA4.1^+^, IgM^hi^, CD23^−^
T2 transitional	B220^+^, AA4.1^+^, IgM^hi^, CD23^+^
T3 transitional	B220^+^, AA4.1^+^, IgM^lo^, CD23^+^
MZB	B220^+^, AA4.1^−^, CD23^lo^, CD21^hi^, CD1d^hi^
FoB	B220^+^, AA4.1^−^, CD23^hi^, CD21^lo^, CD1d^lo^
